# Editorial: Genome-Wide Analyses of *Pectobacterium* and *Dickeya* Species

**DOI:** 10.3389/fpls.2022.855262

**Published:** 2022-03-03

**Authors:** Mohammad Arif, Robert Czajkowski, Toni A. Chapman

**Affiliations:** ^1^Department of Plant and Environmental Protection Sciences, University of Hawaii at Manoa, Honolulu, HI, United States; ^2^Laboratory of Biologically Active Compounds, Intercollegiate Faculty of Biotechnology UG and MUG, University of Gdansk, Gdańsk, Poland; ^3^Biosecurity and Food Safety, NSW Department of Primary Industries, Elizabeth Macarthur Agricultural Institute (EMAI), Menangle, NSW, Australia

**Keywords:** pectinolytic bacteria, soft rot and blackleg disease, *Dickeya*, *Pectobacterium*, genome-wide, comparative genomics

*Pectobacterium* and *Dickeya*, emerging pathogens and key genera included in the Soft Rot Pectobacteriaceae family (SRP; formerly known as pectinolytic *Erwinia* spp.) (Adeolu et al., 2016), are among the top 10 bacterial plant pathogens that limit crop yields and threaten global food security worldwide (Mansfield et al., 2012). Species within both genera are globally distributed (Mansfield et al., 2012; Ma et al., 2019; Boluk et al., 2021) and cause significant damage to both monocots and dicots, particularly to potato with potential global losses in production (Agrios, 2006), and an estimated cost of US$50–100 million annually in vegetables, fruits, and ornamental plants (Perombelon and Kelman, 1980; Pérombelon, 2002; Ma et al., 2007). Species in both genera have been isolated from infected plant tissues, soil, and water (Glasner et al., 2008; Hugouvieux-Cotte-Pattat et al., 2019; Oulghazi et al., 2019) as well as from alternative non-agricultural plant hosts (Fikowicz-Krosko et al., 2017; Fikowicz-Krosko and Czajkowski, 2018). The pectinolytic bacteria rapidly adapt to new hosts, raising serious concerns for potential damage to new crops (Boluk et al., 2020, 2021; Klair et al., 2021).

Recently, several new *Pectobacterium* and *Dickeya* species were reported; *Pectobacterium* is currently divided into 19 recognized species with the addition of *P. parvum* in 2020 (Pasanen et al., 2020), and *Dickeya* is divided into 12 species, including the recent addition of *D. oryzae* (Wang et al., 2020).

High levels of virulence associated with these SRPs involves the secretion of plant cell wall degrading enzymes (PCWDEs) primarily through a type II secretion system (T2SS), enabling them to digest their hosts more extensively than any other microbes in both field and storage conditions (Hugouvieux-Cotte-Pattat et al., 2014; Li et al., 2018; Arizala and Arif, 2019; Fan et al., 2020). Pathogenicity determinants play a significant role in host adaptation and virulence (Boluk et al., 2021). PCWDEs and other virulence factors, such as the type III effector protein DspE and necrosis inducing protein Nip, are used to macerate plant tissue and promote plant cell death, providing nutrients for the multiplication and colonization of these necrotrophic pathogens (Kim et al., 2011; Babujee et al., 2012; Charkowski et al., 2012; Haque et al., 2017; Fan et al., 2020). The T1SS, T2SS, T6SS, some PCWDEs and proteases, the ECA cluster, achromobactin, flagellar genes, single virulence locus, the *pilW* and *pilABC* genes, Flp/Tad, carotovoricin, DsbA oxidoreductase, and the majority of virulence regulators were anticipated critical genes/gene clusters for all *Pectobacterium* species, while T3SS, T4SS, T5SS, phytotoxins, type IV pilus, capsular polysaccharide, lipopolysaccharides, exopolysaccharides, iron uptake systems, phenazine, carbapenem, and colicin-like bacteriocins were observed in some species. Differences among antimicrobial compounds and toxin and antitoxin systems surrounding the CRISPR-Cas systems were observed in others (Glasner et al., 2008; Charkowski et al., 2012; Arizala and Arif, 2019; Przepiora et al., 2022).

Despite the severe disease impact of *Pectobacterium* and *Dickeya* species, not many comprehensive studies of SRPs have been conducted to ascertain evolutionary relationships among strains or the role of horizontal gene transfer (HGT) in speciation and adaptation to a new niche. Likewise, the impact of phages and prophages on the ecology and virulence of *Pectobacterium* and *Dickeya* still needs to be assessed in detail to understand the role of these virus-bacteria interactions in natural and agricultural settings (Czajkowski, 2016, 2019).

Rapidly developing sequencing technology (Illumina, Ion Torrent, Pacific Biosciences and Oxford Nanopore) has led to a sharp decrease in sequencing costs and has enabled genome sequencing of plant bacterial strains on a vast scale. Genomics analyses play a dominant role in elucidating bacterial taxonomy, phylogeny, and evolutionary biology and provide insights into distinct niche adaptation (McAdam et al., 2014; Cai et al., 2018; Arizala and Arif, 2019). Comparative genomics of recently evolved or emerging pathogens provide insights into the regions/islands acquired by closely related strains, enabling them to adapt to a new host/environment (Zhang et al., 2020). The number of new “omics datasets—such as genome sequencing, RNA-seq, pan-genomics, and metabolomics—are increasing rapidly. New bioinformatics pipelines and software have also become available, and with the increasing numbers of datasets and bioinformatics pipelines and software, we can accelerate the progress made in comparative and functional bacterial genomics (Chen et al., 2017; Karp et al., 2019).

In this special issue, six articles were published. Czajkowski et al. in their article “Genome-wide identification of *Dickeya solani* transcriptional units upregulated in response to plant tissues from a crop-host *Solanum tuberosum* and a weed-host *Solanum dulcamara*”, identified 210 mutant of *D. solani* IPO2222 exhibited plant tissue-dependent expression. The selected 13 genes were differentially expressed in potato (*Solanum tuberosum*) and/or *Solanum dulcamara* (bittersweet nightshade) stem, leaf, and root tissues. These results imply that necrotrophic bacterium *D. solani* can recognize its hosts during the early stages of infection and modify its behavior accordingly. In the next article “The PhoPQ two-component system is the major regulator of cell surface properties, stress responses, and plant-derived substrate utilization during development of *Pectobacterium versatile*-host plant pathosystems” by Kravchenko et al. it was revealed that PhoP, part of PhoPQ two compartment system, regulates at least 115 genes involved in degradation, transport, and metabolism of plant-derived carbon sources, bacterial cell envelope, and stress resistance, and concluded that PhoPQ is a crucial system regulating multiple virulence-related genes controlling the development of *P. versatile*-host plant pathosystem. Article “*Pectobacterium brasiliense* 1692 chemotactic responses and the role of methyl-accepting chemotactic proteins in ecological fitness” by Tanui et al. identified 34 methyl-accepting chemotactic proteins (MCPs) in *P. brasiliense* Pb 1692. Four out of 34 MCPs were further characterized and found that these MCPs contribute toward the biology and fitness of Pb 1692 during potato infection. Pun et al. in their article “Phloretin, an apple phytoalexin, affects the virulence and fitness of *P. brasiliense* by interfering with quorum-sensing” described that biofilm formation, secretion of plant cell wall-degrading enzymes, and production of acyl–homoserine lactone (AHL) signaling molecules were significantly inhibited by exposing *P. brasiliense* to phloretin, and impaired virulence mechanisms. The results support that phloretin inhibits Expl activity. Genomic biology of two unique strains of *Dickeya zeae* was described in the article “Genomic and phenotypic biology of novel strains of *D. zeae* isolated from pineapple and taro in Hawaii: insights into genome plasticity, pathogenicity, and virulence determinants” by Boluk et al.. The analyses revealed truncated type III and IV secretion systems (T3SS and T4SS) in the taro strain. Both strains, from pineapple and taro, however, were pathogenic, lacking the zeamine biosynthesis gene cluster, a key player in virulence in other *Dickeya* spescies. In the last article “Transcriptome analysis revealed overlapping and special regulatory roles oVf RpoN1 and RpoN2 in motility, virulence, and growth of *Xanthomonas oryzae* pv. *oryzae*” by Yu et al. it was found that deletion of *rpoN1* or *rpoN2* in *X. oyzae* pv. *oryzae* led to significant disfunction of bacterial swimming motility, flagellar assembly, and virulence, and identified 127 overlapping differentially expressed genes (DEGs) regulated by both RpoN1 and RpoN2.

In conclusion, this special issue compiled research articles covering comparative and functional genomics analysis of SRP bacteria. The articles published in this issue added scientific knowledge to fill information gaps related to pathogenicity determinants, genetic exchange, and evolution of this devastating group of pathogens, and enhanced our ability to combat soft rot diseases that unequivocally impact food security.

## Author Contributions

All authors listed have made a substantial, direct, and intellectual contribution to the work and approved it for publication.

## Funding

MA acknowledges the support by NIGMS of the National Institutes of Health under award number P20GM125508. RC acknowledges the grant support of NCN OPUS 13 (2017/25/B/NZ9/00036) from the National Science Center, Poland (Narodowe Centrum Nauki, Polska). TC acknowledges the support by the NSW Department of Primary Industries, Australia.

## Author Disclaimer

The content is solely the responsibility of the authors and does not necessarily represent the official views of the funding agencies.

## Conflict of Interest

The authors declare that the research was conducted in the absence of any commercial or financial relationships that could be construed as a potential conflict of interest.

## Publisher's Note

All claims expressed in this article are solely those of the authors and do not necessarily represent those of their affiliated organizations, or those of the publisher, the editors and the reviewers. Any product that may be evaluated in this article, or claim that may be made by its manufacturer, is not guaranteed or endorsed by the publisher.

## References

[B1] Adeolu M. Alnajar S. Naushad S. Gupta R. S. (2016). Genome-based phylogeny and taxonomy of the 'Enterobacteriales': proposal for Enterobacterales ord. nov. divided into the families *Enterobacteriaceae, Erwiniaceae* fam. nov., *Pectobacteriaceae* fam. nov., *Yersiniaceae* fam. nov., *Hafniaceae* fam. nov., *Morganellaceae* fam. nov., and *Budviciaceae* fam. nov. Int. J. Syst. Evol. Microbiol. 66, 5575–5599. 10.1099/ijsem.0.001485 27620848

[B2] Agrios G. N.. (2006). Bacterial Soft Rots, 5th Edn. San Diego, CA: Academic Press.

[B3] Arizala D. Arif M. (2019). Genome-wide analyses revealed remarkable heterogeneity in pathogenicity determinants, antimicrobial compounds, and CRISPR-Cas systems of complex phytopathogenic genus *Pectobacterium*. Pathogens. 8, 247. 10.3390/pathogens8040247 PMC6963963 31756888

[B4] Babujee L. Apodaca J. Balakrishnan V. Liss P. Kiley P. J. Charkowski A. O.. (2012). Evolution of the metabolic and regulatory networks associated with oxygen availability in two phytopathogenic enterobacteria. BMC Genom. 13, 110. 10.1186/1471-2164-13-110 PMC3349551 22439737

[B5] Boluk G. Arizala D. Dobhal S. Zhang J. Hu J. Alvarez A. M.. (2021). Genomic and phenotypic biology of novel strains of *Dickeya zeae* isolated from pineapple and taro in Hawaii: insights into genome plasticity, pathogenicity, and virulence determinants. Front. Plant Sci. 12, 663851. 10.3389/fpls.2021.663851 34456933 PMC8386352

[B6] Boluk G. Arizala D. Ocenar J. Mokwele J. Silva J. Dobhal S.. (2020). First report of *Pectobacterium brasiliense* causing soft rot on *Brassica oleracea* var. *sabellica* L. in Hawaii, United States. Plant Dis. 2721. 10.1094/PDIS-04-20-0701-PDN

[B7] Cai H. Bai Y. Guo C. (2018). Comparative genomics of 151 plant-associated bacteria reveal putative mechanisms underlying specific interactions between bacteria and plant hosts. Genes Genom. 40, 857–864. 10.1007/s13258-018-0693-1 30047115

[B8] Charkowski A. Blanco C. Condemine G. Expert D. Franza T. Hayes C.. (2012). The role of secretion systems and small molecules in soft-rot Enterobacteriaceae pathogenicity. Annu. Rev. Phytopathol. 50, 425–449. 10.1146/annurev-phyto-081211-173013 22702350

[B9] Chen I. A. Markowitz V. M. Chu K. Palaniappan K. Szeto E. Pillay M.. (2017). IMG/M: integrated genome and metagenome comparative data analysis system. Nuc. Acids Res. 45, D507–D516. 10.1093/nar/gkw929 27738135 PMC5210632

[B10] Czajkowski R.. (2016). Bacteriophages of soft rot *Enterobacteriaceae*-a minireview. FEMS Microbiol. Lett. 363, fnv230. 10.1093/femsle/fnv230 26626879

[B11] Czajkowski R.. (2019). May the phage be with you? Prophage-like elements in the genomes of soft rot *Pectobacteriaceae: Pectobacterium* spp. and *Dickeya* spp. Front. Microbiol. 10, 138. 10.3389/fmicb.2019.00138 30828320 PMC6385640

[B12] Fan J. Ma L. Zhao C. Yan J. Che S. Zhou Z.. (2020). Transcriptome of *Pectobacterium carotovorum* subsp. *carotovorum* Pcc51 infected in calla plants *in vivo* highlights a spatiotemporal expression pattern of genes related to virulence, adaptation, and host response. Mol Plant Pathol. 21, 871–889. 10.1111/mpp.12936 32267092 PMC7214478

[B13] Fikowicz-Krosko J. Czajkowski R. (2018). Systemic colonization and expression of disease symptoms on bittersweet nightshade (*Solanum dulcamara*) infected with a GFP-tagged *Dickeya solani* IPO2222 (IPO2254). Plant Dis. 102, 619–627. 10.1094/PDIS-08-17-1147-RE 30673477

[B14] Fikowicz-Krosko J. Wszalek-Rozek K. Smolarska A. Czajkowski R. (2017). First report of isolation of soft rot *Pectobacterium carotovorum* subsp. carotovorum from symptomless bittersweet nightshade occuring in rural area of Poland. J. Plant Pathol. 99:294. 10.4454/jpp.v99i1.3772

[B15] Glasner J. D. Marquez-Villavicencio M. Kim H.-S. Jahn C. E. Ma B. Biehl B. S.. (2008). Niche-specificity and the variable fraction of the *Pectobacterium* pan-genome. Mol. Plant Microbe Interact. 21, 1549–1560. 10.1094/MPMI-21-12-1549 18986251

[B16] Haque M. M. Oliver M. M. H. Nahar K. Alam M. Z. Hirata H. Tsuyumu S. (2017). CytR homolog of *Pectobacterium carotovorum* subsp. *carotovorum* controls air-liquid biofilm formation by regulating multiple genes involved in cellulose production, c-di-GMP signaling, motility, and type Ill secretion system in response to nutritional and environmental signals. Front. Microbiol. 8, 972. 10.3389/fmicb.2017.00972 PMC5449439 28620360

[B17] Hugouvieux-Cotte-Pattat N. Condemine G. Shevchik V. E. (2014). Bacterial pectate lyases, structural and functional diversity: bacterial pectate lyases. Environ. Microbiol. 6, 427–440. 10.1111/1758-2229.12166 25646533

[B18] Hugouvieux-Cotte-Pattat N. Jacot-des-Combes C. Briolay J. (2019). *Dickeya lacustris* sp. nov., a water-living pectinolytic bacterium isolated from lakes in France. Int. J. Syst. Evol. Microbiol. 69, 721–726. 10.1099/ijsem.0.003208 30724725

[B19] Karp D. P. Ivanova N. Krummenacker M. Kyrpides N. Latendresse M. Midford P.. (2019). A comparison of microbial genome web portals. Front. Microbiol. 10, 208. 10.3389/fmicb.2019.00208 PMC6395428 30853946

[B20] Kim H. S. Thammarat P. Lommel S. A. Hogan C. S. Charkowski A. O. (2011). *Pectobacterium carotovorum* elicits plant cell death with DspE/F but the *P. carotovorum* DspE does not suppress callose or induce expression of plant genes early in plant-microbe interactions. Mol. Plant Microbe Int. 24, 773–786. 10.1094/MPMI-06-10-0143 21469936

[B21] Klair D. Silva J. Arizala D. Boluk G. Dobhal S. Ahmad A. A.. (2021). First Report of *Pectobacterium brasiliense* causing soft rot on mizuna (*Brassica rapa* var. *japonica*) in the United States. Plant Dis. 4149. 10.1094/PDIS-03-21-0644-PDN

[B22] Li X. Ma Y. Liang S. Tian Y. Yin S. Xie S.. (2018). Comparative genomics of 84 *Pectobacterium* genomes reveals the variations related to a pathogenic lifestyle. BMC Genom. 19, 889. 10.1186/s12864-018-5269-6 PMC6286560 30526490

[B23] Ma B. Hibbing M. E. Kim H. S. Reedy R. M. Yedidia I. Breuer J.. (2007). Host range and molecular phylogenies of the soft rot entero-bacterial genera *Pectobacterium* and *Dickeya*. Phytopath. 97, 1150–1163. 10.1094/PHYTO-97-9-1150 18944180

[B24] Ma X. Perna N. T. Glasner J. D. Hao J. Johnson S. Nasaruddin A. S.. (2019). Complete genome sequence of *Dickeya dianthicola* ME23, a pathogen causing blackleg and soft rot diseases of potato. Microbiol. Resour. Announc. 8, e01526–e01518. 10.1128/MRA.01526-18 30801063 PMC6376422

[B25] Mansfield J. Genin S. Magori S. Citovsky V. Sriariyanum M. Ronald M.. (2012). Top 10 plant pathogenic bacteria in molecular plant pathology. Mol. Plant Pathol. 13, 614–629 10.1111/j.1364-3703.2012.00804.x 22672649 PMC6638704

[B26] McAdam P. R. Richardson E. J. Fitzgerald J. R. (2014). High-throughput sequencing for the study of bacterial pathogen biology. Curr. Opin. Microbiol. 19, 106–113. 10.1016/j.mib.2014.06.002 25033019 PMC4150483

[B27] Oulghazi S. Pédron J. Cigna J. Lau Y. Y. Moumni M. Van Gijsegem F.. (2019). *Dickeya undicola* sp. nov., a novel species for pectinolytic isolates from surface waters in Europe and Asia. Int. J. Syst. Evol. Microbiol. 69, 2440–2444. 10.1099/ijsem.0.003497 31166160

[B28] Pasanen M. Waleron M. Schott T. Cleenwerck I. Misztak A. Waleron K.. (2020). *Pectobacterium parvum* sp. nov., having a Salmonella SPI-1-like Type III secretion system and low virulence. Int. J. Syst. Evol. Microbiol. 70, 2440–2448. 10.1099/ijsem.0.004057 32100697 PMC7395620

[B29] Pérombelon M. C. M.. (2002). Potato diseases caused by soft rot erwinias: an overview of pathogenesis. Plant Pathol. 51, 1–12. 10.1046/j.0032-0862.2001.Shorttitle.doc.x

[B30] Perombelon M. C. M. Kelman A. (1980). Ecology of the Soft Rot Erwinias. Annu. Rev. Phytopath. 18, 361–387. 10.1146/annurev.py.18.090180.002045

[B31] Przepiora T. Figaj D. Bogucka A. Fikowicz-Krosko J. Czajkowski R. Hugouvieux-Cotte-Pattat N.. (2022). The periplasmic oxidoreductase DsbA is required for virulence of the phytopathogen *Dickeya solani*. Int. J. Mol. Sci. 23, 697. 10.3390/ijms23020697 35054882 PMC8775594

[B32] Wang X. He S.-W. Guo H.-B. Han J.-G. Thin K. K. Gao J.-S.. (2020). *Dickeya oryzae* sp. nov., isolated from the roots of rice. Int. J. Syst. Evol. Microbiol. 70, 4171–4178. 10.1099/ijsem.0.004265 32552985

[B33] Zhang J. Arif M. Shen H. Hu J. Sun D. Pu X.. (2020). Genomic divergence between *Dickeya zeae* strain EC2 isolated from rice and previously identified strains, suggests a different rice foot rot strain. PLoS ONE 15, e0240908. 10.1371/journal.pone.0240908 33079956 PMC7575072

